# Sexual violence and pregnancy-related physical symptoms

**DOI:** 10.1186/1471-2393-12-83

**Published:** 2012-08-11

**Authors:** Mirjam Lukasse, Lena Henriksen, Siri Vangen, Berit Schei

**Affiliations:** 1 Department of Public Health and General Practice at the Faculty of Medicine, The Norwegian University of Science and Technology (NTNU), Håkon Jarls gate 11, N-7489, Trondheim, Norway; 2 Department of Health, Nutrition and Management, Faculty of Health Sciences, Oslo and Akershus University College of Applied Sciences, Postboks 364 Alnabru, N-0614, Oslo, Norway; 3 Section of Obstetrics at the Division of Obstetrics and Gynecology, Oslo University Hospital, Postboks 4950 Nydalen, N-0424, Oslo, Norway; 4 National Resource Centre for Women’s Health at the Division of Obstetrics and Gynecology, Oslo University Hospital, Postboks 4950 Nydalen, N-0424, Oslo, Norway; 5 Department of Gynecology at the Women’s Clinic, St. Olavs Hospital,Trondheim University Hospital, Postbox 3250, Sluppen, N-7006, Trondheim, Norway

**Keywords:** Sexual violence, Rape, Pregnancy, Physical complaints

## Abstract

**Background:**

Few studies have investigated the impact of sexual violence on health during pregnancy. We examined the association between sexual violence and the reporting of physical symptoms during pregnancy.

**Methods:**

A population-based national cohort study conducted by The Norwegian Mother and Child Cohort study (MoBa) collected data from pregnant women through postal questionnaires at 17 and 32 weeks gestation. Three levels of sexual violence were measured: 1) mild (pressured into sexual relations), 2) moderate (forced with violence into sexual relation) and 3) severe (rape). Differences between women reporting and not reporting sexual violence were assessed using Pearson’s *X*^2^ test and multiple logistic regression analyses.

**Results:**

Of 78 660 women, 12.0% (9 444) reported mild, 2.8% (2 219) moderate and 3.6% (2 805) severe sexual violence. Sexual violence was significantly associated with increased reporting of pregnancy-related physical symptoms, both measured in number of symptoms and duration/degree of suffering. Compared to women not reporting sexual violence, the probability of suffering from ≥8 pregnancy-related symptoms estimated by Adjusted Odds Ratio (AOR) was 1.49 (1.41–1.58) for mild sexual violence, 1.66(1.50–1.84) for moderate and 1.78 (1.62–1.95) for severe. Severe sexual violence both previously and recently had the strongest association with suffering from ≥8 pregnancy-related symptoms, AOR 6.70 (2.34–19.14).

**Conclusion:**

A history of sexual violence is associated with increased reporting of pregnancy-related physical symptoms. Clinicians should consider the possible role of a history of sexual violence when treating women who suffer extensively from pregnancy-related symptoms.

## Background

Sexual violence comprises a wide range of sexual violent acts. According to the World Health Organization (WHO) sexual violence includes any sexual act or attempt to obtain a sexual act using coercion [1]. Coercion may involve physical force, psychological intimidation and threats [1]. Sexual violence includes rape, traditionally defined as vaginal, anal or oral sexual intercourse obtained through force or threat of force [1,2]. However, more recently the recognition has developed that coercion may not always be present or essential for sexual violence to occur. Therefore newer definitions of rape and sexual violence have replaced the term coercion by lack of consent, thus including sexual violence that occurs through the inability to give consent, for example due to intoxication [3,4].

The lifetime prevalence of sexual violence among nationally representative samples of women in the USA ranges from 18.0% for rape [5,6] to 27.2% for unwanted sexual contact [6]. Population-based studies from Australia, Sweden and Norway report a prevalence ranging from 8.1 to 13.3% [7-10], while WHO in their multi-country study reported a lifetime prevalence ranging from 6.2% in Japan to 58.6% in Ethiopia [11]. Prevalence estimates vary, depending on the population studied, investigation methods used, response rate achieved and how rape was defined [12].

Risk factors for experiencing sexual violence are young age, high-risk behavior including alcohol/substance misuse, and other violence such as intimate partner or domestic violence [5,13,14]. A history of sexual violence has been associated with a wide range of psychological and physical complaints as well as medical diagnoses including post-traumatic stress disorder, depression, anxiety disorders, eating disorders, somatization disorders, chronic pain such as headaches, abdominal pain, fibromyalgia and pelvic pain, gastro-intestinal symptoms and sexually transmitted diseases [8,15-18]. Women who have experienced sexual violence are more likely to report poor quality of health compared to women without a history of abuse [15-18]. Women’s pre-pregnancy health, health perception and negative health behaviors are likely to continue during pregnancy thus affecting pregnancy and pregnancy outcome [9].

Even though an uncomplicated pregnancy is generally considered to be a state of health rather than disease, it is frequently accompanied by so called “minor symptoms” of pregnancy, such as nausea and vomiting, tiredness, backache, heartburn, constipation, vaginal discharge, leg cramps, edema, headache, Braxton Hicks contractions, urinary incontinence, pelvic girdle relaxation, and urinary tract infections [19-21]. These symptoms are primarily the result of physiological changes caused by pregnancy and usually have no bearing on the outcome of pregnancy [19,21]. They are subjective and may be difficult to substantiate objectively.

As far as we know only two previous studies have investigated the association between sexual abuse and pregnancy-related physical symptoms [22,23]. In both studies the sexual abuse was limited to abuse during childhood. The aim of our study was to investigate if a history of sexual violence is associated with the number of pregnancy-related symptoms women report. In addition we wanted to explore whether women with a history of sexual violence suffered to a greater extent from the reported symptoms, compared to women without such a history.

## Material and methods

### Design and population

The Norwegian Mother and Child Cohort Study (MoBa study) conducted by the Norwegian Institute of Public Health is a nationwide cohort study [24]. This large-scale study was not based on any single or even set of hypotheses, but aimed to estimate the association between a large number of exposures and outcomes [24]. A large group of researchers was involved in the planning of the study and developing the questionnaires. In order to include many possible relevant exposures some validated instruments were included in a shortened and modified version while other questions were specifically developed for this study. The study collected data from pregnant women using three extensive questionnaires.

From 1999 to 2009 the majority of all pregnant women were invited to participate through a postal invitation. Of all the women giving birth in Norway during the inclusion period, approximately 40% participated in the MoBa study, of which 92% completed both questionnaires used in our data analysis. This present study is a cross-sectional study using data from the cohort study and included 92 838 pregnancies, comprising women who returned both the first and the third questionnaire (Q1 at 16–20 and Q3 at 30–34 weeks gestation). We subsequently excluded 13 475 pregnancies of women who had participated twice or more (i.e. only a woman’s first pregnancy was included) and 703 women who had not responded to any of the questions on sexual abuse in Q1, leaving a total of 78 600 women for analyses. Informed consent was obtained from each participant. The Regional Committee for Medical Research Ethics (Regional Komité for Forskningsetikk Helseregion II, Ref.SAFH95/313RTL) and the Norwegian Data Inspectorate approved the study. The safety of participants potentially involved in ongoing abuse was ensured by the following measures. Recruiting hospitals were able to care for participants and if necessary refer them to other institutions thereby ensuring the safety of participants potentially involved in ongoing abuse. The aim of the MoBa study is, as the invitation to participate explained, to study factors influencing general and obstetric health. The questions on violence and abuse were therefore not expected, either by the participants or their partners. In addition, these questions were not immediately apparent as they were placed at the end of a long questionnaire.

### Variables

**Figure 1:**
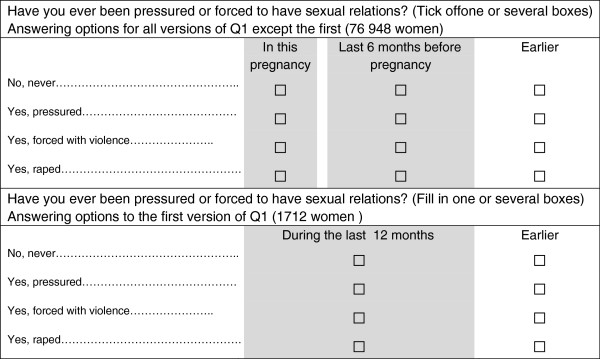
Questions about sexual violence in Q1, questionnaire 16–20 weeks gestation, in Norwegian mother and child cohort study.

The exposure variable came from Q1, measuring sexual violence at 3 levels of severity. Women were asked if they ever had been pressured or forced into sexual relations. Answering options were: 1) No, never; 2) Yes, pressured (mild); 3) Yes, forced with violence (moderate); 4) Yes, raped (severe). A positive answer was defined as having experienced sexual violence. Women with more than one positive answer were classified according to the most severe level reported. For each of the 4 answering options women could indicate when the sexual violence had occurred (Figure 1). The format of this question was changed slightly after version 1 of Q1 (Figure 1). All versions of Q1 had the answering option “earlier”, which we coded as previously. The answering option “during the last 12 months”, from version 1 of Q1, was coded as recent sexual violence, as were the options “during this pregnancy” and “during the last 6 months before pregnancy” from all subsequent versions of Q1.

The physical complaints were derived from both Q1 and Q3, except for Braxton Hicks contractions and leg cramps, which were only reported in Q3. Women indicated if they were “not at all”, “a little bothered” or “bothered a lot” by Braxton Hicks contractions. For the remaining physical symptoms, women reported how many 4-week periods they were bothered by each symptom. The number of 4-week periods women could tick off, varied from 8 for most symptoms (total of 32 weeks starting from 0–4 weeks of pregnancy) to 5 for leg cramps (only Q3, started from 13–16 weeks of pregnancy).

Information on adult physical violence was taken from Q1 and consisted of a positive answer as to whether women as adults had experienced being slapped, hit, kicked or otherwise physically assaulted. This question and the ones on physical violence and emotional abuse from Q3 are modified questions derived from the Norvold Abuse Questionnaire (NorAq) [25]. 

Child physical violence from Q3 consisted of a positive answer to the question “Have you experienced physical violence before the age of 18?”. Emotional abuse either as a child (<18 years) or as an adult (≥18 years), also from Q3, was a positive answer to one or both of two descriptive questions: 1) Have you experienced anybody, systematically and over a long period of time, trying to repress, degrade or humiliate you? 2) Have you experienced anybody threatening to hurt you or someone close to you? In Q3 women were asked if they had been pressured into sexual acts/activities either as a child (<18 years) or as an adult (≥18 years). The sexual violence reported in Q1 could very well, but not necessarily, be that reported in Q3. As the question on sexual violence is more detailed in Q1 and as its wording is less likely to include non-contact sexual abuse we selected our exposure variable from Q1. To differentiate between these similar variables from Q1 and Q3, the term sexual abuse is used for Q3 and sexual violence for Q1.

The Hopkins Symptom Checklist using 5 items (SCL-5), from Q3, was used to measure mental distress, using a 2.0 cut-off point as suggested by Strand et al. [26]. The SCL-5 had been translated into Norwegian and validated in Norway [27]. Socio-demographic and other characteristics such as age, education, parity, civil status, occupation, consumption of alcohol or smoking during pregnancy, height and pre-pregnancy weight were derived from Q1. Any report of smoking or alcohol use during pregnancy was coded as positive for these variables.

### Statistical analyses

Frequency analyses were used to quantify the prevalence of the different levels of sexual violence at different time periods. Cross-tabulation and Pearson’s chi-square test were used to study percentages and assess differences in the prevalence of demographic and other characteristics for women reporting sexual violence compared to women not reporting sexual violence.

There were no significant differences for missing data by level of severity of sexual violence. The prevalence of missing data was 2.1% for BMI, 3.7% for education, 0.5% for civil status, and 0.7% for smoking during pregnancy. Missing data for the SCL-5 (3.2%) was replaced by the series mean. Sensitivity analyses comparing the total score of SCL-5 before and after imputation showed no significant differences. The results of the logistic regression analyses remained qualitatively the same when performed with complete exclusion of missing data compared to using imputed missing data for SCL-5.

In a clinical setting without routine enquiry about a history of violence, women rarely inform their doctor or midwife about such a history [10,28]. However, women who complain “excessively” of pregnancy-related physical symptoms may be noticed. We therefore aimed at analyses which would identify those women from the total sample. We did this by defining a cut-off for the number of 4-week periods women were suffering from the different symptoms using the 90^th^ percentile of the distribution for the whole sample. The same procedure was used to define the cut-off for the number of pregnancy-related physical symptoms. This identified women who reported 8 or more symptoms.

The questions enquiring about sexual violence in Q1 and sexual abuse in Q3 are not the same, yet very similar. This explains why among the 9 114 women reporting childhood and/or adult sexual abuse in Q3, 7 577 (83.1%) also reported any sexual violence in Q1. Testing for collinearity between sexual violence reported in Q1 and sexual abuse reported in Q3 resulted in a Pearson’s correlation coefficient of 0.605, well above the generally accepted cut-off of <0.4 for entering as a covariate in the regression analyses. Due to the overlap and collinearity shown, sexual abuse from Q3 was not entered in the regression analyses models.

Binary logistic regression analyses were performed to estimate the crude and adjusted OR and 95% CI for the association between the different levels of severity of sexual violence and the 90^th^ percentile of the number of 4-week periods of suffering for each pregnancy-related physical symptom. Age was considered the only true confounding factor and controlled for in all the models [29]. The literature shows that co-occurrence of violence is common [17]. In order to estimate the independent association between the sexual violence and the reporting of pregnancy-related physical symptoms, we adjusted for the other types of violence and abuse reported, as well as age in all adjusted models, provided there were enough cases. Mental distress has been associated with sexual violence and we considered it to be a mediating factor [17]. In *Model 1* we adjusted for age and other types of violence and abuse. In *Model 2* we additionally adjusted for mental distress to estimate the importance of this mediating factor. Finally, in *Model 3*, we added the a priori selected covariates: pre-pregnancy BMI, parity, smoking and alcohol consumption in early pregnancy to those included in *Model 2*. The covariates added to *Model 3* are all well-known factors associated with a history of abuse and violence [30-34]. They are also known risk factors for several of the pregnancy-related physical symptoms [35-38]. *Model 3* investigates the importance of these factors. The comparison group for all analyses was women not reporting sexual violence. All analyses were two-sided at α 0.05 and conducted with the statistical program SPSS version 19.0.

## Results

Twelve percent (9 444) of the women reported mild sexual violence, 2.8% (2 219) moderate and 3.6% (2 805) severe (Table 1). Of the 14 468 women who reported sexual violence ever, 94.4 % reported having experienced this only previously, 1.6% only recently and 4% both recently and previously.

**Table 1:** Prevalence of sexual violence in the Norwegian mother and child cohort study, N = 78 660

***Level of severity of sexual violence***	***Only recently***	***Only previously***	***Both previously and recent***	***Ever***
	**n (%)**	**n (%)**	**n (%)**	**n (%)**
Mild	195 (0.2)	8 837 (11.2)	412 (0.5)	9444 (12.0)
Moderate	15 (0.0)	2200 (2.8)	4 (0.0)	2219 (2.8)
Severe	54 (0.1)	2737 (3.5)	14 (0.0)	2805 (3.6)
Any	264 (0.3)	13 774 (17.5)	430 (0.55)	14 468 (18.4)

Women reporting a history of sexual violence were significantly younger, more often unemployed and less frequently living with a partner (Table 2). In addition they more frequently reported smoking and alcohol consumption during early pregnancy, a BMI ≥30 and mental distress (Table 2). Women reporting sexual violence were significantly more likely to report other types of violence and abuse, both as a child and as an adult (Table 2). One thousand five hundred and thirty seven (1 537) women reported sexual abuse in Q3 without reporting sexual violence in Q1, of these, 1 134 reported childhood sexual abuse and 429 adult sexual abuse.

**Table 2:** Characteristics of women without and with a mild, moderate or severe history of sexual violence in the mother and child cohort study, N = 78 660 (column %)

	***No sexual violence***	***Mild sexual violence***	***Moderate sexual violence***	***Severe sexual violence***
	**n = 64 192%**	**n = 9 444%**	**n = 2 219%**	**n = 2 805%**
Age*				
<19	1.1	1.9	2.5	6.1
20–25	16.6	18.7	18.7	26.7
26–31	48.8	43.3	42.3	38.5
32–37	29.2	30.8	30.7	23.7
≥38	4.2	5.3	5.7	4.9
Education*				
Primary	1.9	3.3	5.7	9.7
Secondary	34.4	40.4	45.8	54.3
Higher ≤4 years	40.6	36.4	30.7	24.3
Higher >4 years	23.1	19.9	17.8	11.7
Occupation*				
Employed	82.8	76.3	70.9	62.4
Student	9.5	12.7	14.0	16.2
Not employed	7.7	11.0	15.1	21.4
Civil status*				
Married/cohabitant	97.3	94.2	92.3	88.9
Other	2.7	5.8	7.7	11.1
Smoking during pregnancy*	7.1	11.8	16.5	22.4
Alcohol during pregnancy*	11.3	14.9	14.6	12.5
Parity*				
Nulliparous	55.4	53.1	51.4	55.4
Multiparous	44.6	46.9	48.6	44.6
Pre-pregnancy BMI*				
<20	12.5	12.7	13.3	14.3
20–24.9	56.7	55.9	52.4	48.4
25–29.9	21.6	21.4	22.0	23.3
≥30	9.2	10.0	12.2	13.9
Mental distress*	5.1	12.2	14.5	20.9
Adult physical violence*	9.2	30.7	47.0	53.0
Child physical violence*	2.8	11.0	22.8	34.6
Adult emotional abuse*	12.1	31.4	40.4	44.7
Child emotional abuse*	10.6	23.9	33.0	39.3
Adult sexual abuse Q3*	0.7	20.1	36.1	40.6
Child sexual abuse Q3*	1.8	20.1	40.6	52.8

* P < 0.001 using Pearson *X*^2^.

All through pregnancy the proportion of women suffering from pregnancy-related physical symptoms was significantly higher among women with a history of sexual violence compared to women without such a history. Already at 0–4 weeks, 45% of the women with a history of severe sexual violence reported suffering one or more pregnancy-related physical symptoms, compared to 33.3% for women not reporting sexual violence (P > 0.001). At 21–24 weeks and 29–32 weeks, the proportion of women reporting suffering one or more symptoms had risen to 80.5% and 79.2% respectively for women reporting a history of sexual violence compared to 71.7% and 72.5% for those without a history of sexual violence (P > 0.001). Severe sexual violence was significantly associated with an increased duration of pregnancy-related symptoms (Table 3). An increasing level of severity of sexual violence was associated with an increasing probability of suffering for a longer period of time for the majority of the physical symptoms (Table 3). An increasing level of severity of sexual violence was also associated with an increasing probability of reporting ≥ 8 symptoms (Table 4). Compared to women not reporting sexual violence, those reporting mild sexual violence were nearly twice as likely to report ≥8 symptoms, crude OR 1.95 (1.85–2.06), while women reporting severe sexual violence were three times as likely to report ≥8 symptoms, crude OR 3.09 (2.84–3.36) (Table 4). These associations were attenuated, but still significant, when controlling for factors such as age and other types of violence and abuse (Table 4). In a dose–response fashion, the more types of violence and abuse to which women were exposed, the more likely they were to report ≥8 physical symptoms (not in the tables). The unadjusted OR for women with a history of any sexual violence (including all levels of severity) reporting ≥8 pregnancy-related physical symptoms was 2.24 (2.15–2.35). For women reporting two types, such as both any sexual violence and adult physical violence, the crude OR was 2.70 (2.54–2.87), and for any sexual violence and adult emotional abuse the crude OR was 2.86 (2.68–3.04). For women reporting three types: any sexual violence as well as adult physical violence and adult emotional abuse, the crude OR was 3.18 (2.93–3.44). Having experienced any sexual violence both recently and previously resulted in a stronger association with ≥ 8 symptoms, crude OR 3.07 (2.57–3.66), than only previously, crude OR 2.22 (2.12–2.34), and only recently, crude OR 1.73 (1.25–2.40) (not in the tables). A similar pattern was evident for each of the different levels of severity of sexual violence when analyzed separately (Table 5).

**Table 3:** The duration/degree of pregnancy-related symptoms by level of sexual violence, percentages and adjusted † OR, N = 78 660

	No. of 4-week period(s) *	***No sexual violence *n = 64 192**	***Mild sexual violence *n = 9 444**		***Moderate sexual violence *n = 2 219**		***Severe sexual violence *n = 2 805**	
		**%**	**%**	**AOR (95% CI)**	**%**	**AOR (95% CI)**	**%**	**AOR (95% CI)**
1. Backache	≥5	9.2	13.6	1.29 (1.21–1.38)	15.4	1.32 (1.17–1.49)	18.9	1.48 (1.33–1.65)
2. Tiredness	≥5	13.2	18.1	1.27 (1.20–1.35)	18.8	1.23 (1.10–1.38)	21.8	1.45 (1.31–1.61)
3. Constipation	≥5	9.8	13.5	1.31 (1.22–1.40)	14.7	1.36 (1.20–1.54)	13.0	1.20 (1.06–1.36)
4. Pelvic girdle relaxation	≥4	12.3	16.6	1.23 (1.16–1.31)	18.3	1.25 (1.11–1.40)	21.2	1.40 (1.26–1.55)
5. Heartburn	≥4	11.7	15.2	1.21 (1.14–1.29)	18.1	1.39 (1.24–1.56)	20.1	1.54 (1.39–1.71)
6. Nausea and vomiting	≥3	10.8	12.3	1.26 (1.19–1.34)	14.6	1.27 (1.14–1.43)	17.7	1.66 (1.50–1.83)
7. Edema	≥2	11.6	15.8	1.21 (1.14–1.29)	18.6	1.33 (1.18–1.49)	20.9	1.47 (1.32–1.62)
8. Candidiasis	≥2	12.1	15.1	1.22 (1.14–1.30)	14.9	1.15 (1.02–1.30)	14.1	1.07 (0.95–1.20)
9. Pruritus gravidarum	≥1	13.6	19.0	1.30 (1.23–1.38)	21.1	1.34 (1.20–1.49)	24.1	1.50 (1.36–1.66)
10. Leukorrhea	≥1	7.2	10.7	1.37 (1.27–1.48)	11.6	1.39 (1.21–1.60)	11.1	1.26 (1.11–1.44)
11. Headache	≥2	15.0	20.9	1.27 (1.20–1.34)	23.4	1.33 (1.20–1.48)	26.7	1.48 (1.35–1.62)
12. Urinary Tract Infection	≥1	9.3	11.2	1.09 (1.01–1.17)	13.0	1.18 (1.03–1.35)	13.2	1.11 (0.98–1.25)
13. Urine incontinence	≥1	11.7	17.1	1.33 (1.25–1.41)	17.4	1.22 (1.09–1.37)	19.1	1.35 (1.21–1.50)
14. Leg cramps	≥3	11.1	13.0	1.14 (1.05–1.20)	13.7	1.14 (1.00–1.29)	13.1	1.05 (0.93–1.06)
15. Braxton Hicks contractions	bothered a lot **	12.5	17.0	1.26 (1.19–1.34)	18.3	1.27 (1.14–1.43)	23.0	1.66 (1.50–1.83)

† Adjusted for age, child physical violence, adult physical violence, child emotional abuse and adult emotional abuse. Comparison group is the group of women not reporting sexual violence.* Number of 4-week periods women indicated having suffered from this symptom, maximum possible number of 4-week periods for symptom 1–10 was 8, for symptom 11– 3 it was 6 and for symptom14 it was 5. ** Question without 4-week periods option.

**Table 4:** The Odds Ratios (crude and adjusted) for having ≥8 pregnancy-related symptoms, n = 11 532

***Type of violence and abuse***	***≥8 symptoms***	***Crude OR***	***Adjusted OR Model 1***	***Adjusted OR Model 2***	***Adjusted OR Model 3***
	**n**	**Crude OR (95% CI)**	**AOR (95% CI)**	**AOR (95% CI)**	**AOR (95% CI)**
Sexual violence					
Mild	2 057	1.95 (1.85–2.06)	1.49 (1.41–1.58)	1.44 (1.37–1.53)	1.44 (1.36–1.53)
Moderate	595	2.57 (2.33–2.83)	1.66 (1.50–1.84)	1.61 (1.46–1.79)	1.60 (1.44–1.78)
Severe	859	3.09 (2.84–3.36)	1.78 (1.62–1.95)	1.68 (1.53–1.84)	1.64(1.49–1.81)
Adult physical violence	2 806	2.20 (2.10–2.31)	1.35 (1.28–1.43)	1.31 (1.24–1.38)	1.30 (1.22–1.37)
Child physical violence	1 255	2.57 (2-39–2.75)	1.31 (1.21–1.42)	1.27 (1.17–1.38)	1.22 (1.13–1.33)
Adult emotional abuse	3 200	2.28 (2.17–2.38)	1.74 (1.65–1.83)	1.68 (1.60–1.77)	1.48 (1.40–1.56)
Child emotional abuse	2 641	2.12 (2.02–2.23)	1.53 (1.45–1.62)	1.48 (1.40–1.57)	1.70 (1.62–1.80)

Comparison group is the group of women not reporting sexual violence.

*Model 1*: adjusted for age and other types of abuse.

*Model 2*: adjusted for age, other types of violence and abuse and mental distress.

*Model 3*: adjusted for age, other types of violence and abuse, mental distress, BMI, smoking and alcohol consumption and parity.

**Table 5:** The crude and adjusted OR for having ≥8 pregnancy-related symptoms by timing and level of severity of sexual violence

	***≥8 symptoms *n = 11 532**	***Crude *OR (95% CI)**	***Adjusted *OR (95% CI)**
Mild sexual violence			
Only previously	1902	1.92 (1.82–2.03)	1.45 (1.37–1.54)*
Only recently	39	1.75 (1.23–2.49)	1.71 (1.20–2.43)**
Both previously and recently	116	2.74 (2.21–3.41)	2.04 (1.63–2.55)*
Moderate sexual violence			
Only previously	591	2.57 (2.33–2.83)	1.58 (1.42–1.76)*
Only recently	2	1.08 (0.24–4.77)	Too few to adjust
Both previously and recently	2	7.00 (0.99–49.72)	Too few to adjust
Severe sexual violence			
Only previously	844	3.12 (2.87–3.40)	1.68 (1.53–1.86)*
Only recently	8	1.22 (0.57–2.58)	1. 18 (0.56–2.50)**
Both previously and recently	7	7.00 (2.46–19.97)	6.70 (2.34–19.14)**

Comparison group is the group of women not reporting sexual violence.

* adjusted for age and other types of abuse and violence ** adjusted for age only.

## Discussion

Eighteen percent of the women in our study reported having experienced any sexual violence. Women with a history of sexual violence suffered from more pregnancy-related physical symptoms, to a greater extent and for a longer time, compared to women who did not report such a history. Having experienced sexual violence both recently and previously resulted in a stronger association with suffering from ≥8 symptoms than sexual violence experienced only previously or only recently.

Our study has several strengths. Firstly, the large sample size gives robust results and allowed considerable adjustment for confounding and mediating factors. In particular, we were able to assess the independent association for sexual violence by controlling for other types of abuse and violence. Secondly, selection bias in relation to the exposure is unlikely as women consenting to participate were not expecting questions on sexual violence. Thirdly, the sample being population-based suggests that our results are generalizable for the Norwegian setting and very likely beyond. However, the low response rate causes concern. We lack information on why women did not participate. Participation involved considerable effort and had no immediate benefits for the women taking part. Nilsen et al. [39] compared participants in the MoBa study to all women giving birth in Norway. They found a strong under-representation of the youngest women (<25 years), those living alone, mothers with more than two previous births and smokers [39]. Despite this, no statistically relative differences in association measure were found between participants and the total population regarding the eight association measures they tested [39]. They therefore concluded that even though the prevalence of both exposure and outcome may be different in the MoBa study compared to the entire pregnant population in Norway, the estimates of association can still be valid [39].

A major limitation of our study is the lack of use of a validated instrument for measuring sexual violence. The Norwegian questionnaire uses the term “seksuell omgang”. The most correct translation for this term in English is “sexual relations”, not “intercourse” as written in the English version of the questionnaire translated for the benefit of researchers (not used by participants). The term “sexual relations” is not precise and can include other acts besides intercourse. However, the term points towards physical contact as opposed to non-contact sexual abuse. All three answering options suggest that the sexual relations were unwanted. Our results show an increase in strength of associations from mild to severe sexual violence, which suggests that the study participants also have interpreted these different levels as increasing levels of severity. Our study does not include questions about the timing, frequency or perpetrator of the sexual violence. Information about such factors could shed important light on our findings. For example, we do not know if the prevalence of sexual violence and associations investigated differ significantly for a known compared to unknown perpetrator.

Seventeen percent of the women reporting sexual abuse in Q3 did not report sexual violence in Q1. The majority of these women reported abuse before the age of 18 in Q3. A reasonable explanation for this lack of overlap is that the questions, although similar, are not exactly the same. The questions in Q1 point towards physical-contact sexual violence. The question in Q3 could more easily be interpreted as also including non-contact sexual abuse. For some of those reporting sexual abuse as an adult in Q3 but not in Q1, a possible but unlikely explanation could be that the abuse happened after answering Q1.

Another limitation of our study is the lack of knowledge of the severity of the pregnancy-related physical symptoms, except for the Braxton Hicks contractions. Women were asked if and when they suffered from the pregnancy-related physical symptoms, but not how much. However, even if the women had been asked how badly they suffered from these physical symptoms, the measurement would have remained subjective. Most of the pregnancy-related symptoms are rarely substantiated objectively, as they generally cause no concern regarding the outcome of the pregnancy (e.g. heartburn, constipation, leg cramps, backache), while some symptoms may lead to further investigation to rule out pathology (e.g. edema, pruritis gravidarum, headache, leukorrhea).

Our study, like most others investigating the impact of sexual violence, relies on retrospective self-reporting with the risk of recall bias [40]. Self-reporting begins with the individual perceiving and storing the experience as a memory of sexual violence. Next the study questions have to trigger the participant recall of the event. Studies have shown that the methodology used, i.e. the number of questions asked, the phrasing and the context in which the questions appear, influence the rates of self-reported sexual violence [41,42]. Lastly women have to be willing to disclose their experience [40,41]. Women in our study were sent the questionnaires by post, and if the perpetrator of the unwanted sexual relations was their present partner, fear of retribution resulting from the partner reading their responses may well have stopped disclosure. This could also be one of the reasons why the prevalence of recent sexual violence was so low compared to previously experienced sexual violence. However, our prevalence of recent sexual violence of 0.8% (0.3% only recent and 0.5% both previously and recent) is very similar to that of 1% reported in the first national population based study of violence among Norwegian women [7].

Our lifetime prevalence of ever having experienced sexual violence (18%) is exactly the same as the prevalence reported in the general population (not college students) from a nationwide study in the USA by Kilpatrick et al. [5]. It is difficult to compare our estimates of prevalence of sexual violence with other Norwegian studies due to methodological differences [7,43,44]. A Nordic study on the prevalence of different types of abuse among patients visiting gynecology clinics reported 6.4% prevalence for women ≥18 for severe sexual abuse which compares well to our study when we combine the prevalence for moderate and severe sexual violence [10].

A history of sexual violence was associated with the reporting of other types of violence and abuse, particularly during adulthood. The co-occurrence increased with the increasing level of severity of sexual violence (Table 2). This pattern suggests that sexual violence in our study was part of intimate partner violence or domestic violence. This finding agrees with other research reporting that in the majority of sexual violence cases, the perpetrator is known to the victim. The most common perpetrator of sexual violence occurring in childhood is the father, stepfather or another relative. For adult sexual violence, it is a partner or former partner [5,9]. Co-occurrence of multiple forms of violence and adult re-victimization as suggested in this study are well documented findings [7,16,45,46].

No other studies have investigated the association between a history of any lifetime sexual violence and pregnancy-related physical symptoms. However, other studies have noted the association between sexual violence and a range of somatic health problems in predominantly non-pregnant women [7,15-18,44,45]. Two studies among pregnant women showed a significant association between childhood sexual abuse and physical symptoms and complaints [22,23]. A Swedish study of a general population of pregnant women with the primary purpose to determine the prevalence of lifetime sexual abuse, reported that such a history was associated with increased reporting of general health problems [9]. They focused on diagnoses and diseases with little attention to symptoms reported by women.

The MoBa study is a large epidemiological study designed to investigate many correlations [24] but not causality. Our study therefore examined the association between different levels of sexual violence and pregnancy-related physical symptoms. We did, however, estimate the effect on the associations for some of the intermediate factors which according to the literature [47-49] are considered to be on the pathway between exposure and outcome by adjusting for them in logistic regression models. Different pathways have been proposed to explain the association between sexual violence and pregnancy-related physical symptoms [47,48]. Some symptoms could be linked to behavioral risk factors, such as obesity and smoking, which are more prevalent among victims of sexual violence and abuse, both in our study and others [48]. In our study, adjusting for these factors did not alter the association considerably. The psychological pathway seems of importance. As with the reporting of most physical complaints, psychological factors may increase the reporting of pregnancy-related physical symptoms [48,50]. This pathway includes conditions such as hyper-vigilance, somatization, anxiety, sleeping difficulties and hostility, and is put forward by several researchers in relation to the experience of physical symptoms and poor self-reported health [15,47-49,51,52]. In addition, some studies suggest that current life stressors increase the rate of health problems more for abused individuals than for those without a history of abuse [53,54]. Pregnancy and the anticipation of childbirth itself have been recognized as possible stressors for most pregnant women, while severe fear of childbirth has been associated with a history of sexual abuse [55-57]. In our study we noticed that the associations changed noticeably when mental distress was entered in the model. Our study, which is based on data from a cohort study, has a cross-sectional design and can therefore not show a causal link between the experience of sexual violence and pregnancy-related physical symptoms. However, in most cases the sexual violence occurred before the pregnancy. This fact and the increased strength of the association with increased severity offer support to a causal association [29].

## Conclusions

We found that women who reported sexual violence suffered longer and from more pregnancy-related physical symptoms compared to women not reporting sexual violence. The symptoms may seem like minor complaints to those who provide health care during pregnancy. However, they may cause women major discomfort and severely affect their well-being during pregnancy. Few women spontaneously disclose their history of violence to health professionals [10,28]. Clinicians should consider the possible role of a history of sexual violence or other abuse when treating women who suffer to a great extent from pregnancy-related physical symptoms.

## Abbreviations

Q1: Questionnaire 1; Q3: Questionnaire 3; AOR: Adjusted Odds Ratio; OR: Odds Ratio; CI: Confidence Interval; NorAq: Norvold Abuse questionnaire; BMI: Body Mass Index; SCL-5: Symptom Check List with 5 items; MoBa: Norwegian Mother and Child Cohort Study; MBRN: Medical Birth Registry of Norway.

## Competing interests

There are no competing interests.

## Authors’ contributions

ML conceived the study, performed the analyses, drafted and corrected the manuscript. LH participated in the statistical analyses and drafting of the manuscript. SV participated in the conception of the study, advised on the statistical analyses and participated in the drafting of the manuscript. BS participated in the conception and design of the study, advised on the statistical analyses and drafted the manuscript. All authors read and approved the final manuscript.

## Pre-publication history

The pre-publication history for this paper can be accessed here:

http://www.biomedcentral.com/1471-2393/12/83/prepub

## References

[B1] Krug EG Dahlberg LL Mercy JA Zwi AB Lozano R World Report on Violence and Health 2002 World Health Organization, Geneva

[B2] Koss MP Gidycz CA Wisniewski N The scope of rape: incidence and prevalence of sexual aggression and victimization in a national sample of higher education students J Consult Clin Psychol 1987 55 162 170 3494755 10.1037//0022-006x.55.2.162

[B3] Zinzow HM Resnick HS Amstadter AB McCauley JL Ruggiero KJ Kilpatrick DG Drug- or alcohol-facilitated, incapacitated, and forcible rape in relationship to mental health among a national sample of women J Interpers Violence 2010 25 2217 2236 10.1177/0886260509354887 20100896 PMC2967593

[B4] U.S.Department of Justice Attorny General Eric Holder Announces Revisions to the Uniform Crime Report's Definition of Rapehttp://www.fbi.gov/news/pressrel/press-releases/attorney-general-eric-holder-announces-revisions-to-the-uniform-crime-reports-definition-of-rape

[B5] Kilpatrick DG Resnick HS Ruggiero KJ Conoscenti LM McCauley J Drug-facilitated, incapacitated, and forcible rape: a national study 2007 National Crime Victims Research Center, Charleston, USA

[B6] Black MC Basile KC Breiding MJ Smith SG Walters ML Merrick MT The National Intimate Partner and Sexual Violence Survey: 2010 Summary Report 2011 National Center for Injury, Prevention and Control

[B7] Neroien AI Schei B Partner violence and health: results from the first national study on violence against women in Norway Scand J Public Health 2008 36 161 168 10.1177/1403494807085188 18519280

[B8] Rees S Silove D Chey T Ivancic L Steel Z Creamer M Lifetime prevalence of gender-based violence in women and the relationship with mental disorders and psychosocial function JAMA 2011 306 513 521 10.1001/jama.2011.1098 21813429

[B9] Stenson K Heimer G Lundh C Nordstrom ML Saarinen H Wenker A Lifetime prevalence of sexual abuse in a Swedish pregnant population Acta Obstet Gynecol Scand 2003 82 529 536 10.1034/j.1600-0412.2003.00111.x 12780423

[B10] Wijma B Schei B Swahnberg K Hilden M Offerdal K Pikarinen U Emotional, physical, and sexual abuse in patients visiting gynaecology clinics: a Nordic cross-sectional study Lancet 2003 361 2107 2113 10.1016/S0140-6736(03)13719-1 12826432

[B11] Garcia-Moreno C Jansen HA Ellsberg M Heise L Watts CH WHO Multi-country Study on Women's Health and Domestic Violence against Women Study Team: Prevalence of intimate partner violence: findings from the WHO multi-country study on women's health and domestic violence Lancet 2006 368 1260 1269 10.1016/S0140-6736(06)69523-8 17027732

[B12] Krebs CP Lindquist CH Warner TD Fisher BS Martin SL Childers JM Comparing sexual assault prevalence estimates obtained with direct and indirect questioning techniques Violence Against Women 2011 17 219 235 10.1177/1077801210397743 21307031

[B13] Avegno J Mills TJ Mills LD Sexual assault victims in the emergency department: analysis by demographic and event characteristics J Emerg Med 2009 37 328 334 10.1016/j.jemermed.2007.10.025 18394848

[B14] Tjaden P Thoennes N Extent, Nature, and Consequences of Rape Victimization: Findings From the National Violence Against Women Survey 2000 National Institute of Justice and the Centres for Disease Control and Prevention

[B15] Zinzow HM Amstadter AB McCauley JL Ruggiero KJ Resnick HS Kilpatrick DG Self-rated health in relation to rape and mental health disorders in a national sample of college women J Am Coll Health 2011 59 588 594 10.1080/07448481.2010.520175 21823953 PMC3206265

[B16] Bonomi AE Anderson ML Rivara FP Thompson RS Health outcomes in women with physical and sexual intimate partner violence exposure J Womens Health 2007 16 987 997 10.1089/jwh.2006.0239 17903075

[B17] Ellsberg M Jansen HA Heise L Watts CH Garcia-Moreno C WHO Multi-country Study on Women's Health and Domestic Violence against Women Study Team: Intimate partner violence and women's physical and mental health in the WHO multi-country study on women's health and domestic violence: an observational study Lancet 2008 371 1165 1172 10.1016/S0140-6736(08)60522-X 18395577

[B18] Pikarinen U Saisto T Schei B Swahnberg K Halmesmaki E Experiences of physical and sexual abuse and their implications for current health Obstet Gynecol 2007 109 1116 1122 10.1097/01.AOG.0000259906.16474.86 17470592

[B19] Enkin M Keirse MJNC Neilson J Crowther C Duley L Hodnett E Enkin M, Keirse MJNC, Neilson J, Crowther C, Duley L, Hodnett E Unpleasant symptoms in pregnancy A Guide to effective care in pregnancy and childbirth 2000 3 Oxford University Press, Oxford 95 107

[B20] Draper L Pregnant women in the workplace: distinguishing between normal and abnormal physiologic changes AAOHN J 6 A.D 2006 54 217 223 10.1177/216507990605400505 16729658

[B21] Freeman WS Common complaints in pregnancy Med Times 1980 108 7 13 6453264

[B22] Grimstad H Schei B Pregnancy and delivery for women with a history of child sexual abuse Child Abuse Negl 1999 23 81 90 10.1016/S0145-2134(98)00113-6 10075195

[B23] Lukasse M Schei B Vangen S Øian P Childhood Abuse and Common Complaints in Pregnancy Birth 2009 36 190 199 10.1111/j.1523-536X.2009.00323.x 19747265

[B24] Magnus P Irgens LM Haug K Nystad W Skjaerven R Stoltenberg C Cohort profile: the Norwegian Mother and Child Cohort Study (MoBa) Int J Epidemiol 2006 35 1146 1150 10.1093/ije/dyl170 16926217

[B25] Swahnberg IMK Wijma B The NorVold Abuse Questionnaire (NorAQ): validation of new measures of emotional, physical, and sexual abuse, and abuse in the health care system among women Eur J Public Health 2003 13 361 366 10.1093/eurpub/13.4.361 14703325

[B26] Strand BH Dalgard OS Tambs K Rognerud M Measuring the mental health status of the Norwegian population: a comparison of the instruments SCL-25, SCL-10, SCL-5 and MHI-5 (SF-36) Nord J Psychiatry 2003 57 113 118 10.1080/08039480310000932 12745773

[B27] Tambs K Moum T How well can a few questionnaire items indicate anxiety and depression? Acta Psychiatr Scand 1993 87 364 367 10.1111/j.1600-0447.1993.tb03388.x 8517178

[B28] Trautman DE McCarthy ML Miller N Campbell JC Kelen GD Intimate partner violence and emergency department screening: computerized screening versus usual care Ann Emerg Med 2007 49 526 534 10.1016/j.annemergmed.2006.11.022 17276547

[B29] Rothman KJ Epidemiology: an introduction 2002 Oxford University Press, Oxford

[B30] Hillis SD Anda RF Dube SR Felitti VJ Marchbanks PA Marks JS The association between adverse childhood experiences and adolescent pregnancy, long-term psychosocial consequences, and fetal death Pediatrics 2004 113 320 327 10.1542/peds.113.2.320 14754944

[B31] Roche M Moracco KE Dixon KS Stern EA Bowling JM Correlates of intimate partner violence among female patients at a North Carolina emergency department N C Med J 2007 68 89 94 17566552

[B32] Flynn HA Walton MA Chermack ST Cunningham RM Marcus SM Brief detection and co-occurrence of violence, depression and alcohol risk in prenatal care settings Arch Womens Ment Health 2007 10 155 161 10.1007/s00737-007-0188-6 17594132

[B33] Noll JG Zeller MH Trickett PK Putnam FW Obesity risk for female victims of childhood sexual abuse: a prospective study Pediatrics 2007 120 e61 e67 10.1542/peds.2006-3058 17606550

[B34] Nayak MB Lown EA Bond JC Greenfield TK Lifetime victimization and past year alcohol use in a U.S. population sample of men and women drinkers Drug Alcohol Depend 2012 123 213 219 10.1016/j.drugalcdep.2011.11.016 22177898 PMC3322290

[B35] Liang CC Chang SD Lin SJ Lin YJ Lower urinary tract symptoms in primiparous women before and during pregnancy Arch Gynecol Obstet 2012 285 1205 1210 10.1007/s00404-011-2124-2 22042166

[B36] Foxcroft KF Rowlands IJ Byrne NM McIntyre HD Callaway LK Exercise in obese pregnant women: the role of social factors, lifestyle and pregnancy symptoms BMC Pregnancy Childbirth 2011 11 4 10.1186/1471-2393-11-4 21226958 PMC3025919

[B37] Habr F Raker C Lin CL Zouein E Bourjeily G Predictors of gastroesophageal reflux symptoms in pregnant women screened for sleep disordered breathing: A secondary analysis Clin Res Hepatol Gastroenterol 2012 7 May, Epub ahead of publication 10.1016/j.clinre.2012.03.036 22572522

[B38] Meyer LC Peacock JL Bland JM Anderson HR Symptoms and health problems in pregnancy: their association with social factors, smoking, alcohol, caffeine and attitude to pregnancy Paediatr Perinat Epidemiol 1994 8 145 155 10.1111/j.1365-3016.1994.tb00445.x 8047482

[B39] Nilsen RM Vollset SE Gjessing HK Skjaerven R Melve KK Schreuder P Self-selection and bias in a large prospective pregnancy cohort in Norway Paediatr Perinat Epidemiol 2009 23 597 608 10.1111/j.1365-3016.2009.01062.x 19840297

[B40] Cook SL Gidycz CA Koss MP Murphy M Emerging issues in the measurement of rape victimization Violence Against Women 2011 17 201 218 10.1177/1077801210397741 21307030

[B41] Testa M Livingston JA VanZile-Tamsen C The impact of questionnaire administration mode on response rate and reporting of consensual and nonconsensual sexual behavior Psychol Women Q 2005 29 345 352 10.1111/j.1471-6402.2005.00234.x

[B42] Abbey A Parkhill MR Koss MP The effect of frame of reference on responses to questions about sexual assault victimization and perpetration Psychol Women Q 2005 29 364 373 10.1111/j.1471-6402.2005.00236.x PMC4594833 26451071

[B43] Mossige S, Stefansen K Vold og overgrep mot barn og unge 2007 Norsk institutt for forskning om oppvekst, velferd og aldring 20

[B44] Dahle T Aalvik Dalen H Meland E Breidablik HJ Uønskede seksuell erfaringer og helseplager blant ungdom Tidsskrift for Den norske legeforening 2010 130 1912 1916 20930878 10.4045/tidsskr.09.0907

[B45] Campbell J Jones AS Dienemann J Kub J Schollenberger J O'Campo P Intimate partner violence and physical health consequences Arch Intern Med 2002 162 1157 1163 10.1001/archinte.162.10.1157 12020187

[B46] Classen CC Palesh OG Aggarwal R Sexual revictimization: a review of the empirical literature Trauma Violence Abuse 2005 6 103 129 10.1177/1524838005275087 15753196

[B47] Kendall-Tackett KA Inflammation, cardiovascular disease, and metabolic syndrome as sequelae of violence against women: the role of depression, hostility, and sleep disturbance Trauma Violence Abuse 2007 8 117 126 10.1177/1524838007301161 17545569

[B48] Rodgers CS Lang AJ Twamley EW Stein MB Sexual trauma and pregnancy: a conceptual framework J Womens Health (Larchmt) 2003 12 961 970 10.1089/154099903322643884 14709184

[B49] Seng JS A conceptual framework for research on lifetime violence, posttraumatic stress, and childbearing J Midwifery Womens Health 2002 7 337 346 12361345 10.1016/s1526-9523(02)00275-1

[B50] Ursin H Eriksen H Cognitive activation theory of stress, sensitization, and common health complaints Ann N Y Acad Sci 2007 1113 304 310 10.1196/annals.1391.024 17584977

[B51] Kendall-Tackett K Klest B Causal mechanisms and multidirectional pathways between trauma, dissociation, and health J Trauma Dissociation 2009 10 129 134 10.1080/15299730802624510 19333844

[B52] Samelius L Wijma B Wingren G Wijma K Somatization in abused women J Womens Health 2007 16 909 918 10.1089/jwh.2006.0103 17678462

[B53] Cromer KR Sachs-Ericsson N The association between childhood abuse, PTSD, and the occurrence of adult health problems: Moderation via current life stress J Trauma Stress 2006 19 967 971 10.1002/jts.20168 17195985

[B54] Thakkar RR McCanne TR The effects of daily stressors on physical health in women with and without a childhood history of sexual abuse Child Abuse Negl 2000 24 209 221 10.1016/S0145-2134(99)00129-5 10695516

[B55] Lukasse M Vangen S Øian P Schei B Fear of childbirth, women's preference for cesarean section and childhood abuse - a longitudenal study Acta Obstet Gynecol Scand 2010 90 33 40 21275913 10.1111/j.1600-0412.2010.01024.x

[B56] Lukasse M Vangen S Qian P Kumle M Ryding EL Schei B Childhood Abuse and Fear of Childbirth - A Population-Based Study Birth 2010 37 267 274 10.1111/j.1523-536X.2010.00420.x 21083717

[B57] Nerum H Halvorsen L Sorlie T Oian P Maternal request for cesarean section due to fear of birth: Can it be changed through crisis-oriented counseling? Birth 2006 33 221 228 10.1111/j.1523-536X.2006.00107.x 16948722

